# Podophyllotoxin Exposure Affects Organelle Distribution and Functions in Mouse Oocyte Meiosis

**DOI:** 10.3389/fcell.2021.672590

**Published:** 2021-05-19

**Authors:** Ping-Shuang Lu, Lan-Ping Xie, Xiao-Han Kong, Yi Xu, Shao-Chen Sun

**Affiliations:** College of Animal Science and Technology, Nanjing Agricultural University, Nanjing, China

**Keywords:** podophyllotoxin, oocyte, meiosis, organelles, mitochondria

## Abstract

Podophyllotoxin (POD) is one of the most characterized lignans that is commonly found in podophyllum, and its preparations and derivatives are widely used in clinical treatment due to strong antitumor and antivirus activities. POD has been reported for its neurotoxicity, liver toxicity, and potential reproductive toxicity. In the present study, we investigated the effects of POD on the organelles of mouse oocytes during meiosis. Our results showed that exposure to POD significantly reduced the developmental competence of mouse oocytes. Further analysis revealed that the endoplasmic reticulum (ER) failed to accumulate to the spindle periphery, suggesting that POD exposure might affect protein synthesis during oocyte meiotic maturation. Similarly, abnormal Golgi apparatus distribution was found after POD exposure, which could be confirmed by the aberrant localization of Rab11a-related vesicles, indicating that POD induced vesicle-based protein transport disorder. We also found the aberrant accumulation of lysosomes in the cytoplasm of POD-exposed oocytes, which implied that POD might lead to aberrant protein degradation. Moreover, the perinuclear distribution of mitochondria was also significantly disturbed, indicating the mitochondrial dysfunction after POD exposure. In all, our study illustrated that exposure to POD might disrupt protein synthesis, transport, degradation, and ATP production by its effects on the distribution and functions of organelles during mouse oocyte meiotic maturation.

## Introduction

Lignans, as one of the secondary metabolites of plants, are a kind of polyphenols that are widely present in plant tissues such as rhizomes, flowers, leaves, fruits, etc. (Cui et al., 2020). The diversity of the lignan family exhibits a variety of pharmacological activities, such as anti-inflammatory, antioxidant, antitumor, and antivirus effects (Ardalani et al., 2017; Rodríguez-García et al., 2019). Podophyllotoxin (POD) is one of the most characterized lignans that is commonly found in the *Podophyllum* genus, *Dysosma, Diphylleia, Jeffersonia and Catharanthus* etc. (Ardalani et al., 2017). The alcoholic solution of podophyllin was first proven to be an effective treatment for genital warts in 1942 (Giri and Lakshmi Narasu, 2000). However, it has been mostly replaced by POD preparations, 0.5% solution or 0.15% cream, and other alternative therapies due to its high recurrence rate, poor stability, and toxicity (von Krogh and Longstaff, 2001; Wiley et al., 2002; Lacey et al., 2003). In addition, POD is recognized as a potent antitumor factor, and its synthetic derivatives, etoposide and teniposide, have already been used in the clinical treatment of lymphocytic leukemia, certain brain tumors, and lung tumors (Zálešák et al., 2019). Studies have shown that POD is potentially toxic to the nervous and respiratory system since the neurons are swollen and the Nissl substance (RNA/ribosome) reduced significantly after 72-h treatment with POD in adult rats (Chang et al., 1992a). It also has been reported that POD can affect hepatocellular respiration by altering mitochondrial electron flow and inhibiting the synthesis of DNA, RNA, and proteins (Horrum et al., 1986; Yang et al., 1994). Besides, radiolabeled POD reaches a higher concentration in fetal than maternal tissues after giving it to pregnant mice by the oral or intravenous route, and it is still noted to avoid POD therapy during pregnancy or pregnancy planning, indicating the potential effects of POD on reproduction (von Krogh and Longstaff, 2001; Yanofsky et al., 2012). Indeed, the toxicity of POD to the reproductive system especially on germ cells is already reported. In males, exposure to POD causes the mice testicular seminiferous tubules atrophy and degeneration at a certain dose (Chang et al., 1992b). Similarly, POD has harmful effects on rat sperm maturation or fertility due to epididymal epithelial cell apoptosis induced by activating TNF-α and the caspase signaling pathway (Xie et al., 2017). In females, *in vitro* tests confirm that exposure to POD can inhibit microtubule dynamics and meiotic spindle formation, which leads to low oocyte maturing rates and early embryonic developmental competence (Hu et al., 2018). Moreover, porcine oocytes exposed to POD are also subjected to oxidative stress, apoptosis, abnormal spindle formation, and chromosome abnormality (Jiang et al., 2020). However, the toxin effects of POD on the organelle functions of mammalian oocytes have not been studied.

Oocyte quality is important for fertilization in all mammalian species. To ensure the high developmental potential of oocytes, the organelles of oocytes, such as the endoplasmic reticulum (ER), Golgi apparatus, lysosomes, and mitochondria, must have appropriate spatial and temporal dynamics (Sirard et al., 2006). The ER is a complex organelle involved in protein and lipid synthesis, calcium regulation, and interactions with other organelles (Schwarz and Blower, 2016). The Golgi apparatus is the central hub of the secretory pathway where proteins are processed, sorted, and distributed to different destinations (Li et al., 2019). Rab GTPases play vital roles in vesicle formation and trafficking of oocytes (Duan and Sun, 2019), and Rab11 is a modulator of membrane delivery, acting as the intersection between the endocytic and exocytic trafficking pathways (Welz et al., 2014). Previous studies also show that Rab11a-positive vesicles could recruit the myosin Vb and further contribute to oocyte asymmetric spindle orientation during cytokinesis (Holubcová et al., 2013). Lysosomes are membrane-bound organelles that degrade macromolecules through endocytosis, phagocytosis, and autophagy pathways (Luzio et al., 2014). Moreover, mitochondria synthesize adenosine triphosphate (ATP), which is an essential energy currency for many cellular processes (Nunnari and Suomalainen, 2012). Dysfunctional organelles cannot reorganize and store enough mRNAs, proteins, and transcription factors, which are important to oocyte maturation, fertilization, and early embryogenesis (Watson, 2007). It has been found that ER stress has negative effect on oocyte maturation (Lin et al., 2019). Furthermore, intact, functional Golgi membranes are required for germinal vesicle breakdown (GVBD), which is the morphological feature of meiotic resumption (Mao et al., 2014; Pan and Li, 2019). It is also verified that the gradual increase in lysosomal autophagy significantly reduces the fertilization and developmental potential of mature oocytes *in vitro* (McGinnis et al., 2014). Moreover, the stress-induced changes in the mitochondrial function can lead to reduced oocyte maturation (Roth, 2018). Environmental toxins can disrupt oocyte maturation by affecting organelle functions. For example, decabromodiphenyl ethane (DBDPE) is a new brominated flame retardant and an emerging environmental pollutant that induces mitochondrial dysfunction and blocks oocyte maturation (Shi et al., 2021), and a synthetic lactone antibiotic, brefeldin A, has been reported to affect porcine oocyte meiotic maturation by blocking protein transport from the ER to the Golgi apparatus (Cui et al., 2019). Our recent study also shows that citrinin, which is widely found in vegetable-derived foods, impairs the functions of organelles of mouse oocytes and thus affects oocyte maturation (Sun et al., 2020).

In this study, we adopt mouse as the model to explore the potential toxic effects of POD on organelles, including the ER, Golgi apparatus, lysosome, and mitochondria of mammalian oocytes. Our results indicate that POD causes the disruption of the ER, Golgi apparatus, and lysosome distribution in oocytes, which might disturb protein synthesis, transport, and degradation. We also show that POD has adverse effects on mitochondria functions in oocytes. Therefore, our study provides important evidence for the toxicity of POD on organelle functions of mouse oocytes.

## Materials and Methods

### Ethics Statement and Oocyte Culture

Four-week-old female ICR mice were used in the study, and the guidelines of the Animal Research Committee of Nanjing Agriculture University in China were followed. This study was specifically approved by the Animal Research Committee of Nanjing Agriculture University. The mice were kept at a constant temperature of 24°C and with a 12-h light/dark cycle and were provided with adequate food and water. We collected the ovaries to acquire the germinal vesicle stage oocytes in an M2 medium (Sigma, MO, United States). Moreover, the oocytes after washing three times were cultured in an M16 medium for 8.5 h (metaphase I, MI) or 12 h (metaphase II, MII) under paraffin oil at 37°C in 5% CO_2_ atmosphere.

### Antibodies and Chemicals

Rabbit polyclonal anti-Rab11a antibody was from Cell Signaling Technology (Danvers, MA, United States). Alexa Fluor 488 goat anti-rabbit antibody was from Invitrogen (Carlsbad, CA, United States). Hoechst 33342 and all other unstated chemicals and reagents were from Sigma-Aldrich Corp.

### Podophyllotoxin Treatment

Podophyllotoxin (CAS: 518-28-5) was purchased from J&K Corp. (Shanghai, China). It was dissolved in DMSO to 1 μM solution and then diluted in an M16 medium to final concentrations of 0.5 and 1.0 nM. The final concentration of DMSO in the culture medium was less than 0.1%. The oocytes were exposed to different concentrations of POD at 37 °C, 5% CO_2_, and were cultured for 8.5 or 12 h.

### Immunofluorescence Staining and Confocal Microscopy

The oocytes were immobilized in 4% paraformaldehyde for 30 min at room temperature and then permeated with 0.5% Triton X-100 (in PBS) for 20 min. After that, the oocytes were blocked in 1% BSA-supplemented PBS for 1 h. Then we incubated the oocytes with primary antibodies (1:100 for Rab11a) at 4°C overnight or room temperature for 8 h and washed three times with PBS lotion (0.1% Tween and 0.01% Triton X-100) 4 min each time. Next, samples were incubated with the secondary antibodies (Alexa Fluor 488 goat anti-rabbit, 1:100) at room temperature for 1 h. Finally, the oocytes were stained with Hoechst 33342 for 15 min and were sealed and examined by a confocal laser scanning microscope (Zeiss LSM 800 META, Jena, Germany).

### Fluorescence Intensity Analysis

The fluorescence intensity of the samples was analyzed using Image J software. We fixed the control and treated oocytes in a different area on the same glass slide. Moreover, we detected the mean fluorescence intensity per unit area in the region of interest and then statistically analyzed the mean values of the control group and the treatment group.

### Mitochondria and ER Detection

The distribution of mitochondria and the ER in living oocytes was detected by Mito-Tracker Red CMRos (1:600) (Cat# M7512, Invitrogen, Eugene, OR, United States) or ER-Tracker Red (1:500) (C1041 Beyotime Biotechnology Shanghai, China) in an M2 medium at 37°C for 30 min. Then we washed the oocytes three times and scanned the live oocytes with a laser confocal microscope by Zeiss LSM 800 META.

### Golgi Apparatus Detection

For the detection of the Golgi apparatus, we first incubated the oocytes with 1% pronase for 4 min to remove the zona pellucida. Then the living oocytes were incubated with Golgi-Tracker Red (1:100) (C1043 Beyotime Biotechnology Shanghai, China) in an M16 medium at 4°C for 30 min. After washing three times with a fresh culture medium, we incubated the oocytes with M2 at 37°C for 30 min. Finally, we immediately examined the oocytes by a confocal laser-scanning microscope (Zeiss LSM 800 META, Germany).

### Lysosome Detection

Lysosome Red (1:10,000) (C1046 Beyotime Biotechnology Shanghai, China) was used to detect the distribution of lysosome in living oocytes, which were incubated with an M2 medium at 37°C for 30 min. The oocytes were washed three times and then examined by a confocal laser-scanning microscope (Zeiss LSM 800 META, Germany).

### Statistical Analysis

Data were analyzed using the GraphPad Prism 5 software (GraphPad, San Diego, CA, United States), and independent sample *t*-test was used for statistical analysis. We repeated each experiment at least three times. Data were expressed as mean ± SEM. *P* < 0.05 was considered statistically significant.

## Results

### Effects of POD on the Developmental Competence of Mouse Oocytes

**FIGURE 1:**
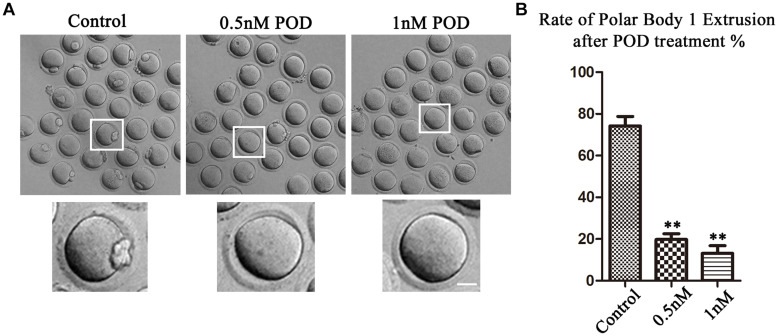
Effects of POD on maturation competence of mouse oocytes. **(A)** The typical picture for the oocyte polar body extrusion in the control and POD-treated group. Bar = 20 μm. **(B)** The rate of polar body extrusion was significantly lower than that in the control group after POD exposure. **P < 0.01.

We first examined the oocyte maturation with increased concentrations (0.5 and 1 nM) of POD after 12 h in culture. As shown in Figure 1A, the majority of the oocytes in the control group could extrude the first polar body (PB1) and reached the stage of metaphase II (MII) (74.18 ± 4.59%, *n* = 146). However, a large number of oocytes failed to extrude PB1 in the POD treatment group, and the maturation rate was significantly reduced compared with the control group (0.5 nM, 19.80 ± 2.72%, *n* = 142, *P* < 0.01; 1 nM, 13.13 ± 3.64%, *n* = 171, *P* < 0.01) (Figure 1B). Therefore, our results showed that POD exposure could reduce the developmental capacity of mouse oocytes, and 0.5 nM POD was selected in our following experiments.

### Effects of POD on ER Distribution in Mouse Oocytes

**FIGURE 2:**
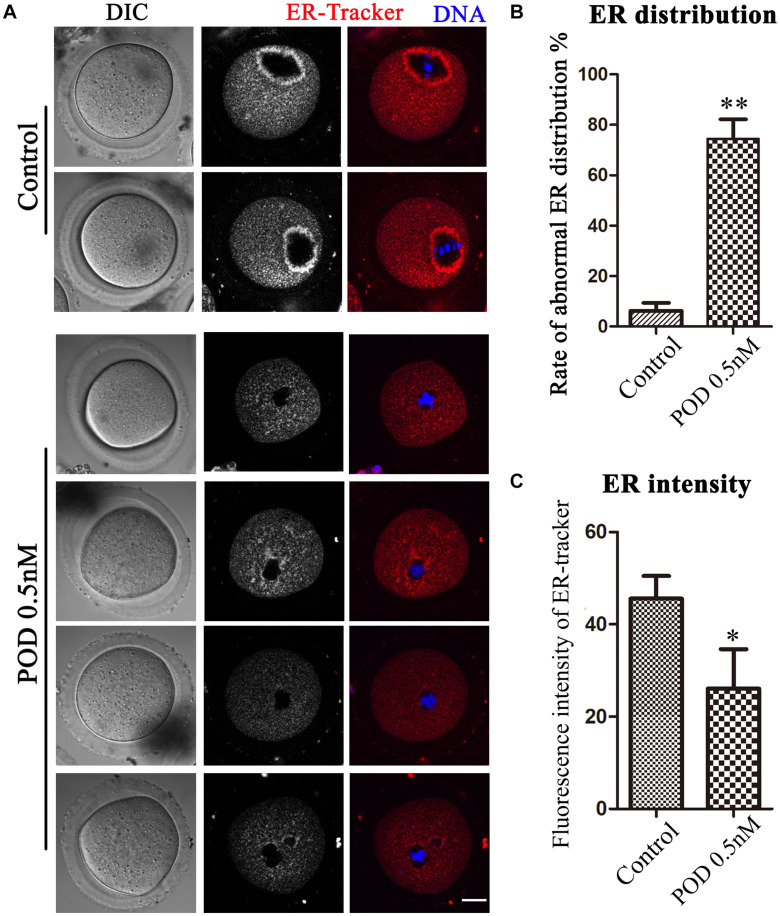
Effects of POD on ER distribution in mouse oocytes. **(A)** The typical picture for the ER distribution in the control and POD-treated groups. Red, ER-Tracker. Blue, DNA. Bar = 20 μm. **(B)** The rate of the abnormal ER distribution was significantly increased in the 0.5 nM POD group compared with the control group. **P < 0.01. **(C)** The fluorescence intensity of ER-Tracker was reduced markedly in the 0.5 nM POD group. *P < 0.05.

The ER is the major site for protein biosynthesis. We then used ER-Tracker to examine the distribution of the ER after POD exposure in mouse oocytes. In the control group, the ER in the MI stage was mainly distributed around the spindle, while oocytes exposed to POD mostly showed a homogenous distribution rather than a perinuclear distribution (Figure 2A). The statistical analysis data also confirmed that the abnormal rate of the ER distribution increased markedly after POD exposure (control group, 6.23 ± 3.14%, *n* = 58; POD group, 74.47 ± 7.72%, *n* = 49, *P* < 0.01) (Figure 2B). To further determine the effects of POD on the ER, we calculated the fluorescence intensities of ER-Tracker and found that it was much weaker in the treatment group than the control oocytes (control group, 45.59 ± 4.88, *n* = 23; POD group, 26.06 ± 8.56, *n* = 22, *P* < 0.05) (Figure 2C). These results demonstrated that POD exposure affected the distribution and function of the ER of mouse oocytes.

### Effects of POD on Golgi Apparatus Distribution in Mouse Oocytes

**FIGURE 3:**
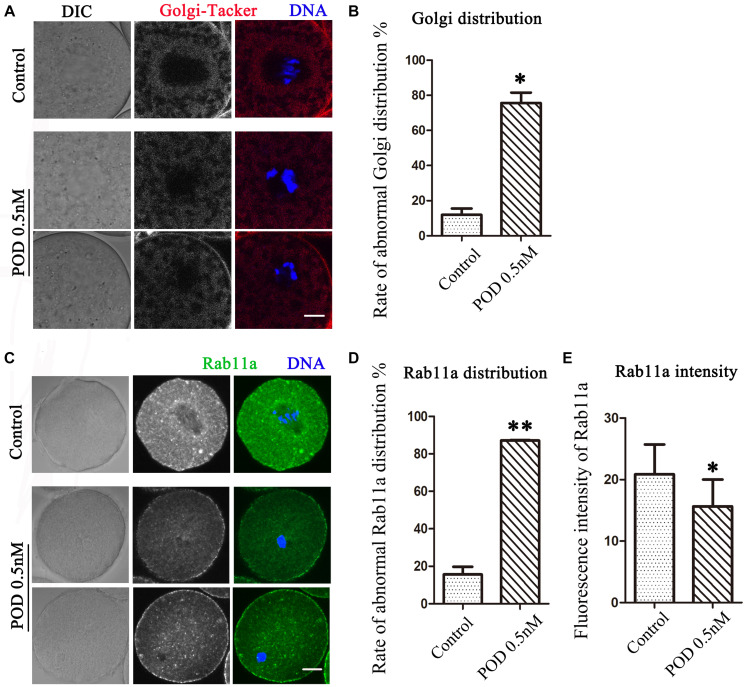
Effects of POD on the Golgi apparatus in mouse oocytes. **(A)** The typical picture for the Golgi distribution after POD exposure in mice. Red, Golgi-Tracker. Blue, DNA. Bar = 10 μm. **(B)** The rate of abnormal Golgi distribution was significantly increased in the 0.5 nM POD group. *P < 0.05. **(C)** The typical picture for Rab11a after POD exposure in mouse oocytes. Green, Rab11a. Blue, DNA. Bar = 20 μm. **(D)** The rate of the abnormal Rab11a distribution was significantly increased in the 0.5 nM POD group. **P < 0.01. **(E)** The relative fluorescence intensity of Rab11a in the 0.5 nM POD group was significantly weaker than the control group *P < 0.05.

The Golgi apparatus, which acts as the docking station for cargo transportations, is closely related to protein synthesis and transportation. We then detected the distribution of the Golgi apparatus by using Golgi-Tracker. As shown in Figure 3A, similar to the ER, the Golgi apparatus of oocytes in the control group was also distributed around the spindle; however, in the treatment group, the Golgi apparatus failed to accumulate to the spindle periphery. In addition, compared with the control group, the abnormal distribution rate of the Golgi apparatus was significantly increased after POD treatment (control group, 11.98 ± 3.56%, *n* = 50; POD group, 75.63 ± 5.88%, *n* = 43, *P* < 0.05) (Figure 3B). Based on the vesicle transport function of the Golgi apparatus, we also examined the distribution of Rab11a, which was related to the vesicle transport. We found that the accumulation of Rab11a around the spindle was reduced in oocytes exposed to POD (Figure 3C). Compared with the control group, the rate of the abnormal distribution of Rab11a in the treatment group was significantly increased (control group, 15.59 ± 4.10%, *n* = 44; POD group, 87.14 ± 0.25%, *n* = 47, *P* < 0.01) (Figure 3D). And the intensity of Rab11a was markedly reduced in the POD-treated oocytes (control group, 20.85 ± 4.86, *n* = 44; POD group, 15.64 ± 7.56, *n* = 47, *P* < 0.05) (Figure 3E). These results suggested that POD exposure disrupted the distribution and function of the Golgi apparatus in mouse oocytes.

### Effects of POD on Lysosome Distribution in Mouse Oocytes

**FIGURE 4:**
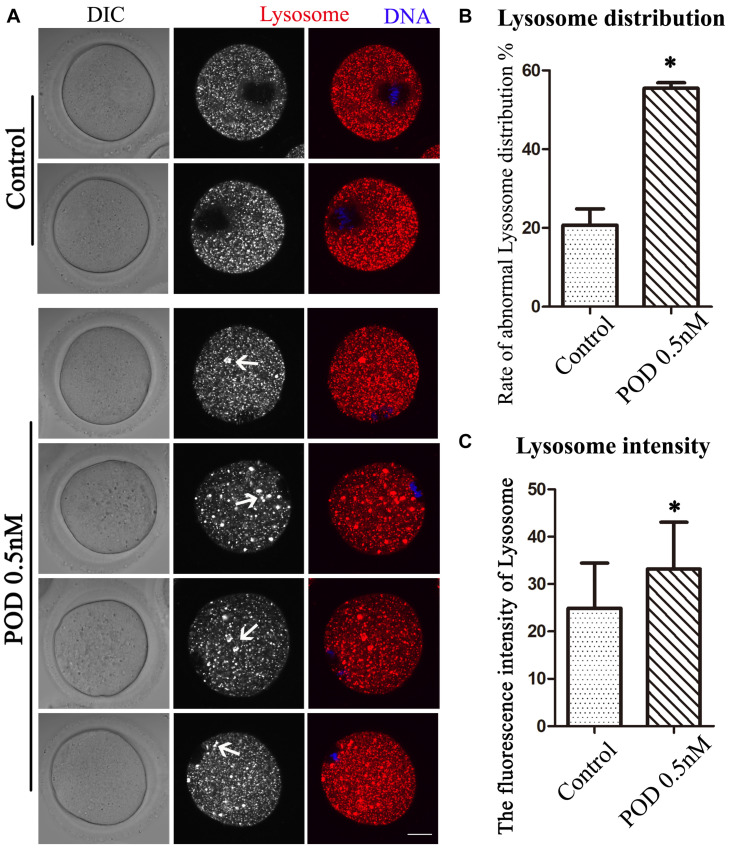
Effects of POD on the lysosome distribution in mouse oocytes. **(A)** The typical picture for the lysosome distribution after POD exposure in mouse oocytes. Red, lysosome. Blue, DNA. Bar = 20 μm. **(B)** The rate of the abnormal lysosome distribution was significantly increased in the 0.5 nM POD group. *P < 0.05. **(C)** The relative fluorescence intensity of lysosome was significantly increased in the 0.5 nM POD group compared with the control group. *P < 0.05.

We also detected lysosomes to further confirm the effects of POD on oocyte organelles. As shown in Figure 4A, we found that the distribution of lysosomes in the control group was homogeneous but agglutinated in the treatment group. In addition, compared with the control group, the rate of the abnormal distribution of lysosome in the treatment group was significantly increased (control group, 20.72 ± 4.18%, *n* = 55; POD group, 55.46 ± 1.43%, *n* = 54, *P* < 0.05) (Figure 4B). Similarly, we also found that the fluorescence intensity of lysosome in the cytoplasm of oocytes was markedly increased in the treatment group compared with the control group (control group, 24.88 ± 16.54, *n* = 40; POD group, 33.17 ± 9.90, *n* = 40, *P* < 0.05) (Figure 4C). These results indicated that POD exposure impaired the function of lysosomes in mouse oocytes.

### Effects of POD on Mitochondria Distribution in Mouse Oocytes

**FIGURE 5:**
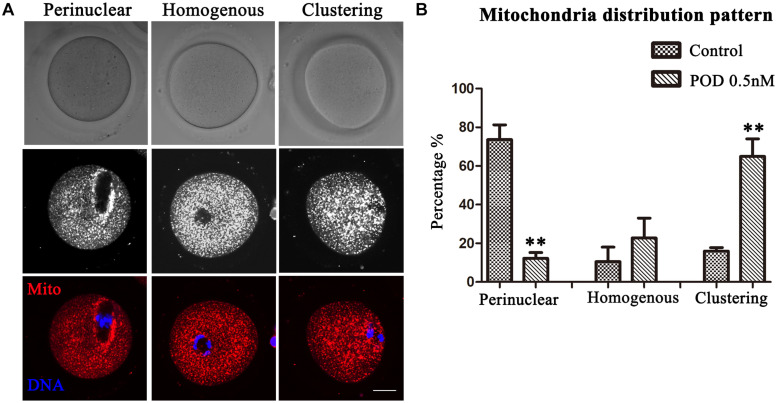
Effects of POD on the mitochondria distribution in mouse oocytes. **(A)** The typical picture for the mitochondria distribution in the control and POD-treated group. Red, Mito-Tracker. Blue, DNA. Bar = 20 μm. **(B)** The perinuclear localization pattern was decreased in the POD-treated group compared with the control group; the clustering localization pattern was increased in the POD-treated group compared with the control group. **P < 0.01.

Mitochondria can provide ATP, which is a key factor to ensure oocyte maturation. Thus, we investigated the mitochondria by Mito-Tracker staining. We classified the mitochondria distribution of MI oocytes into three types: perinuclear phenotype, homogeneous phenotype, and clustering phenotype (Figure 5A). As shown in Figure 5B, a perinuclear distribution (73.61 ± 7.64%), with small proportion of homogenous (10.58 ± 7.47%) and clustering distribution (15.97 ± 1.84%), was observed in the control group (*n* = 44), whereas the proportion of clustering mitochondria (64.99 ± 9.08%, *P* < 0.01) was much higher in the POD-exposed oocytes, showing with a few perinuclear distributions (12.18 ± 3.04%, *P* < 0.01); moreover, there was no significance between the control and the POD group for the homogenous distribution (22.83 ± 10.21%, *P* > 0.1) (*n* = 48). Therefore, these results indicated that POD exposure led to oocyte mitochondrial dysfunction in mice.

## Discussion

With its antivirus and antitumor activities, POD preparations are wildly used in the treatment of genital warts, and its derivatives have also been adopted as a novel natural anticancer agent. POD, a unique lignan, has certain toxic effects on the reproductive system of mammals. In this study, we investigated the distribution of the ER, Golgi apparatus, lysosomes, and mitochondria in mouse oocytes after POD exposure. The results showed that the POD exposure had wide-ranging adverse effects on organelle distribution and functions, which might further lead to declined oocyte quality in mice.

Oocyte maturity and quality are critical for fertilization in mammals. Successful extrusion of PB1 is the last step of oocyte nuclear maturation (Reyes and Ross, 2016). Our results suggest that POD exposure significantly causes declined oocyte quality in mice, showing with a reduction of the PB1 extrusion rate, which is similar to a previous report (Hu et al., 2018). During oocyte maturation, the demand for new proteins that are accomplished by proper protein synthesis, folding, modification, and trafficking is increasing (Guzel et al., 2017). The ER, a multifunctional organelle in eukaryotic cells, is the major store of intracellular calcium and plays a vital role in protein and lipid synthesis (Schwarz and Blower, 2016). To ensure the normal oocyte development, maintaining ER homeostasis is an important issue (Lin et al., 2019). Many studies have reported that the ER is affected after POD derivative exposure, which may imply the potential toxicity of POD on the ER. For example, the epimer of POD can result in esophageal squamous cell carcinoma apoptosis by ER stress and increased ROS level (Kwak et al., 2020). Etoposide, a semisynthetic POD derivative, causes focally extensive dilation of the rough ER in the ganglion cell bodies of female CD-1 mice (Bregman et al., 1994; Wang et al., 2020). Moreover, Ching001 is the structural analog of POD, which partly induces apoptosis *via* the activation of the ER stress signaling pathway, showing with increased expression of p-PREK, p-eIF2α, p-JNK, GADD153, and caspase-4 (Chen et al., 2013). Similarly, our data show that the distribution of the ER is significantly affected after POD exposure, indicating that the protein synthesis is disturbed in mouse oocytes.

The Golgi apparatus acts as a docking station for cargo transportation in which the *cis* face receives the new synthesized molecules from the ER while the *trans* face represents the exit site to a lot of destinations (Wei and Seemann, 2017). Due to its central role, the abnormal structure and function of the Golgi apparatus dramatically influence the cell processes (Kulkarni-Gosavi et al., 2019). Disrupting the Golgi complex by Brefeldin A, a membrane trafficking inhibitor that blocks the anterograde transport of proteins from the ER to the Golgi apparatus, has been suggested to impair oocyte maturation (Moreno et al., 2002; Cui et al., 2019). And the depletion of GM130, a *cis-*Golgi protein, could disturb spindle organization, migration, and asymmetric division during mouse oocyte maturation (Zhang et al., 2011). Moreover, the Golgi apparatus is often affected by the ER due to its role in post-translational modifications and transport of newly synthesized proteins and lipids (Passemard et al., 2019). Our results show that POD led to a failure of spindle periphery accumulation of the Golgi apparatus in mouse oocytes. It is shown that Golgi morphology is changed, and trafficking function is suppressed after exposure to etoposide, which refers to a derivative of POD (Dinsdale et al., 1999; Torii et al., 2020). Besides, aberrant distribution and weak fluorescence intensities of Rab11a are also observed in the POD treatment group oocytes, indicating the decrease in the Rab11a-positive vesicle, which might induce the failure of spindle migration and polar body extrusions (Duan and Sun, 2019). These findings suggest that POD could affect protein and vesicle transportation by Golgi apparatus damage in mouse oocytes.

As protein demand increases, the levels of unfolded or misfolded proteins are enhancing (Guzel et al., 2017), which probably induces ER stress and ultimately causes apoptosis (Lin et al., 2019). Lysosome, the major catabolic center, degrades macromolecules delivered *via* endocytic, phagocytic, and autophagic pathways (Luzio et al., 2014). And this function depends on its more than 50 different hydrolases, which are, respectively, synthesized and transported in the ER and Golgi apparatus (Darios and Stevanin, 2020). The dysfunctional lysosomes can cause various diseases including cancer, neurodegenerative, and autoimmune diseases (Cabukusta and Neefjes, 2018; Darios and Stevanin, 2020). Furthermore, lysosomal dysfunction probably destroys porcine oocyte maturation and developmental capacity by disturbing chromosome and activating autophagy (Miao et al., 2019). POD can inhibit lysosomes to degrade asialo-orosomucoid in rat hepatocytes and prevent the radial redistribution of lysosomes in mouse macrophages (Phaire-Washington et al., 1980; Oka and Weigel, 1983). Our results illustrate that POD inhibits lysosome distribution and function, showing with large lysosomes and strong fluorescence intensities. The enlarged lysosomes likely indicate the occurrence of autophagy, which is considered as a mechanism of defense against oxidative and environmental stress (McGinnis et al., 2014). Therefore, our results demonstrate that POD impairs the function of lysosomes in mouse oocytes.

Mitochondria are the main organelles that produce ATP and are essential to meeting the energy requirements of oocytes. Dysfunction of mitochondria decreases oocyte quality and interferes with embryonic development (Babayev and Seli, 2015). Abnormal structure and function of mitochondria are found in oocytes and cumulus cells from diabetic mice, which may explain the adverse effects of maternal diabetes on embryo development and pregnancy outcomes (Wang et al., 2009, 2010). Our data also show that POD causes significant damage to mitochondria distribution, which may ultimately lead to mitochondria dysfunction in mouse oocytes. Previous studies have shown that POD exposure increases the ROS level and leads to oxidative stress (Jiang et al., 2020), which is interconnected with mitochondrial dysfunction (Willems et al., 2015; Kudryavtseva et al., 2016). We speculate that POD affects the oocyte developmental capacity by inducing mitochondrial damage-mediated insufficient energy supply and oxidative stress. Similarly, it has been reported that POD can induce the change of mitochondrial membrane potential and raise the ROS level in green monkey kidney cells (Li et al., 2013). And the inhibition of coupled and uncoupled respiration of both FAD and NAD-linked substrates is still found in isolated and digitonin-permeabilized hepatocytes after POD exposure (Horrum et al., 1986). These previous findings, together with our work, indicate the general conserved effects of POD on the function of mitochondria among different models. It should be noted that our study is adopted with an *in vitro* model, and whether POD has similar effects on oocytes *in vivo* needs further study.

We speculate that there are two possible causes for the aberrant distribution of organelles in oocytes: one is that POD could prevent the assembly of tubulin into microtubules, which serve as a scaffold for organelle transport and positioning (Ardalani et al., 2017; Meiring et al., 2020); another is that the disrupted spindle structure by microtubule disassembly may affect organelle location and function since they accumulate at the spindle periphery at the MI stage, and POD exposure can disrupt the spindle formation (Coticchio et al., 2015; Hu et al., 2018). However, the deeper explanation for the alteration of the distribution after POD exposure still needs further mechanism study.

In summary, our study illustrates that POD exposure disrupts the distribution and functions of organelles in mouse oocytes, including the ER, Golgi apparatus, lysosome, and mitochondria, which further leads to the defects of oocyte meiotic maturation.

## Data Availability Statement

The original contributions presented in the study are included in the article/supplementary material, further inquiries can be directed to the corresponding author/s.

## Ethics Statement

The animal study was reviewed and approved by Animal Research Committee of Nanjing Agriculture University in China.

## Author Contributions

S-CS designed the experiments. P-SL, L-PX, X-HK, and YX performed the experiments. P-SL, L-PX, and YX contributed to the materials. P-SL and L-PX analyzed the data. P-SL, L-PX, and S-CS wrote the manuscript. All authors approved the submission of the manuscript.

## Conflict of Interest

The authors declare that the research was conducted in the absence of any commercial or financial relationships that could be construed as a potential conflict of interest.

## Abbreviations

POD: podophyllotoxin
GVBD: germinal vesicle breakdown
MI: metaphase I
MII: metaphase II
PB1: first polar body.
